# Wild Birds as Drivers of *Salmonella* Braenderup and Multidrug Resistant Bacteria in Wetlands of Northern Italy

**DOI:** 10.1155/2024/6462849

**Published:** 2024-01-12

**Authors:** Camilla Smoglica, Giulia Graziosi, Damiano De Angelis, Caterina Lupini, Annarita Festino, Elena Catelli, Alberto Vergara, Cristina Esmeralda Di Francesco

**Affiliations:** ^1^Department of Veterinary Medicine, University of Teramo, Piano D'Accio, Teramo 64100, Italy; ^2^Department of Veterinary Medical Sciences, University of Bologna, Ozzano dell'Emilia, Bologna 40064, Italy

## Abstract

In this study, the antimicrobial resistance profiles of bacterial strains obtained from wild avian species recovered in wetlands of Northern Italy were described. Cloacal swabs collected from 67 aquatic birds, hunted or found dead in two private hunting grounds, were submitted to microbiological investigations and antimicrobial susceptibility testing using the Vitek 2 system, while specific PCR protocols were applied to screen for genes associated with the resistance. One hundred fifty-seven bacterial strains were characterized. The most frequent isolates were *Enterococcus faecalis* (36/157; 22.9%) and *Escherichia coli* (23/157; 14.6%). Seventy-seven isolates (77/157; 49%) were resulted resistant to at least one antibiotic, and eight isolates (8/157; 5%) were classified as multidrug resistant bacteria. Resistance for critically important antibiotics (linezolid, vancomycin, carbapenems, third-generation cephalosporins, and fluoroquinolones) was also described. *Salmonella* spp. was obtained from a Eurasian teal (*Anas crecca*), and it was subsequently analyzed by whole genome sequencing, revealing the serovar *Salmonella* Braenderup ST22. The phylogenetic analysis, performed with all ST22 described in 2021 and 2022, placed the strain under study in a large clade associated with human salmonellosis cases. These results suggest that migratory aquatic birds may be considered as relevant carriers of critically important antibiotic resistant bacteria and zoonotic food-borne pathogens potentially able to impact public health.

## 1. Introduction

The concept of One Health has its origins in ancient times. Specifically, the Greek physician Hippocrates emphasized the importance of the environment in ensuring overall health. In the 1800s, the German physician and pathologist Rudolf Virchow laid the foundation for the modern concept of One Health with his definition of the term “zoonosis” [1]. More recently, Calvin Schwabe, a veterinary epidemiologist, emphasized the role of veterinary medicine in ensuring human health with the term “One Medicine,” promoting the awareness that veterinary public health includes wildlife, domestic animals, and humans [1, 2].

In 2004, the Wildlife Conservation Society defined the principles of Manhattan to provide guidance for a global, One World, One Health approach to the prevention of epidemic/epizootic diseases and to maintain biodiversity, which is the foundation of ecosystem health [3].

Indeed, 60% of emerging infectious diseases that are reported globally are zoonoses and have originated in wildlife [4]. In this regard, the antibiotic resistance, defined by the World Health Organization (WHO) as one of the main challenges of the twenty-first century, has been investigated in wildlife, with an annual increase in scientific production of 7% from 1979 to 2019 [5].

In detail, the studies have mainly focused on the antibiotic susceptibility of Gram-negative bacteria such as *Escherichia coli* and *Salmonella* spp. [5]. *E. coli*, being part of the gut microbiota and easily dispersed in different ecosystems, has been widely used as an indicator of antibiotic resistance contamination [5]. Indeed, the first antibiotic-resistant bacterium to be described in a wild animal was an *E. coli* isolated from a pigeon around 1975 [6].

Other bacterial species, such as *Salmonella* spp., have been studied in wildlife as potential food-borne pathogens [5]. Indeed, the first outbreak of salmonellosis in wild birds was described by Wilson and MacDonald in the 1960s and later studies to quantify the risk to humans and domestic animals have been carried out since then [7].

Several free-ranging species have been investigated on this topic of antibiotic resistance, and the most represented group of animals in scientific literature is wild birds [5]. Indeed, the migratory capacity of some species makes them able to create links between areas with different anthropogenic impact exploring long-distance routes [8]. However, few studies that include a phenotypic investigation of antibiotic resistance together with genetic analysis are available for wild aquatic birds. Considering the ecology of waterfowl and the specialization of some species as filter feeders, these animals can come into contact with pathogens present in the environment and with antibiotic-resistant bacteria. Furthermore, hunted aquatic birds may be a human source of these pathogens or resistant bacteria through the consumption of raw or undercooked meat [9, 10].

Given the limited available data, the current study aimed to evaluate the presence of resistant Gram-positive and Gram-negative bacteria and food-borne pathogens, such as *Salmonella* spp., in wild aquatic birds hunted or found dead in wetlands of Northern Italy, where numerous migratory species from different breeding grounds congregate seasonally.

## 2. Materials and Methods

### 2.1. Background and Sample Collection

A total of 67 wild aquatic birds hunted or found dead were included in this study, selected among 124 individuals sampled within the application of the National Avian Influenza (AI) Surveillance Plan 2021 (https://www.izsvenezie.it/documenti/temi/influenza-aviaria/piani-sorveglianza/piano-nazionale-influenza-aviaria-2021.pdf, accessed on October 2021) and the Commission Delegated Regulation (EU) 2020/689. Only individuals sampled within 6 hr from hunting and kept at +4°C or fresh carcasses were included in this study.

**Figure 1:**
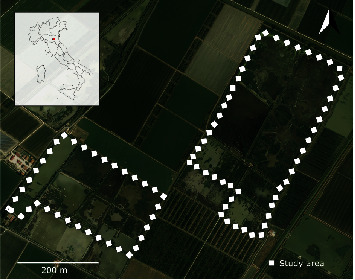
Wetlands in the Bologna province, Emilia-Romagna region, Northern Italy, where hunted waterfowl or wild bird carcasses were collected. Map realized with QGIS software v.3.26 [11].

Sampling activities were conducted from October 2021 to January 2022 in two private hunting grounds (Figure 1) of the province of Bologna, Emilia-Romagna region, Northern Italy. These sites were selected due to numerous overwintering waterfowl species that congregate and intermingle with resident populations of wild birds. AI surveillance activities were performed on the behalf of the Local Health Authority A.U.S.L. of Imola (BO) by sampling birds provided by local hunters or found dead by local ornithologists. Game species were hunted according to the National Hunting Law 157/1992, without the necessity of any additional permits. Cloacal swabs (CS) were collected from each animal using single sterile wooden swabs immersed in BPW, transported at +4°C and immediately processed.

### 2.2. Bacteria Isolation and Antibiotic Susceptibility Test

The isolates were obtained through a nonselective enrichment process of CS for 24 hr at 37°C with buffered peptone water (BPW) (Liofilchem, Italy), followed by subculture using the streak plating technique on MacConkey agar (Liofilchem, Italy) at 37°C for 18–24 hr and Slanetz–Bartley agar (Liofilchem, Italy) at 37°C for 48 hr [12, 13]. After the aforementioned procedures, 1–2 colonies were subcultured to obtain pure cultures for further analysis.

In addition, the detection of *Salmonella* spp. was performed by enrichment and plating out on selective media. Specifically, 100 *μ*l of nonselective enrichment of CS were incubated in 10 ml Rappaport Vassiliadis broth (Liofilchem, Italy) at 41°C for 24 hr. Afterward, aliquots of cultures were spread onto xylose lysine deoxycholate agar (Liofilchem, Italy), and the plates were incubated at 37°C for 24−48 hr [14].

The species identification of colonies and the antimicrobial susceptibility testing were performed using a Vitek 2 system (Biomerieux, France) and MIC Test strip (Liofilchem, Italy). The European Committee on Antimicrobial Susceptibility Testing breakpoints were applied when available; otherwise, CLSI breakpoints were used instead [15, 16].

The antimicrobial susceptibility patterns were determined for a variety of bacteria and antibiotics, with consideration given to the main antimicrobial classes used in veterinary medicine and molecules that are critically important for human medicine, according to European Medicines Agency and WHO guidelines [17, 18]. Gram-negative isolates were tested for 14 antimicrobials: ampicillin, piperacillin/tazobactam, cefotaxime, ceftazidime, ertapenem, extended-spectrum beta-lactamase (ESBL), meropenem, amikacin, gentamicin, ciprofloxacin, tigecycline, tetracyclines, nitrofurantoin, colistin, and trimethoprim/sulfamethoxazole. Susceptibility testing of *Enterococcus* spp. was performed for vancomycin, teicoplanin, linezolid, quinupristin/dalfopristin, gentamicin, kanamycin, streptomycin, ciprofloxacin, levofloxacin, daptomycin, tetracyclines, tigecycline, and nitrofurantoin. The susceptibility of *Streptococcus* spp. to ampicillin, benzylpenicillin, cefotaxime, ceftriaxone, chloramphenicol, clindamycin, erythromycin, gentamycin, levofloxacin, linezolid, moxifloxacin, rifamycin, teicoplanin, tetracycline, tigecycline, and trimethoprim/sulfamethoxazole was evaluated. Finally, susceptibility of *Staphylococcus* spp. was performed for benzylpenicillin, cefoxitin screening, ceftaroline, clindamycin, daptomycin, erythromycin, fusidic acid, gentamicin, levofloxacin, linezolid, mupirocin, oxacillin, rifampicin, teicoplanin, tetracycline, tigecycline, trimethoprim/sulfamethoxazole, and vancomycin.

### 2.3. Whole Genome Sequencing and Phylogenetic Analysis

Considering that *Salmonella* spp. is a zoonotic and indicator bacterium investigated in annual European Reports on antibiotic resistance in humans, animals, and food [19, 20], the *Salmonella* isolate identified in this study was analyzed by whole genome sequencing.

Total genomic DNA from single selected colony was extracted using MagPurix 12A Nucleic Acid Extraction System (Zinexts Life Science Corp., Taipei, Taiwan).

The Nextera XT kit (Illumina, San Diego, CA, USA) was used to prepare the DNA library, and sequences were obtained via the MiSeq instrument (Illumina, San Diego, CA, USA) with a 2 × 300 paired-end run with double indexing. The Bioanalyzer 2100 (Agilent Technologies, Palo Alto, CA) was utilized to validate the library, while the Qubit 2.0 Fluorometer (Invitrogen, Waltham, USA) was used to quantify the starting material and library. All bioinformatics analyses were performed using the designed pipeline on the public ARIES Galaxy server [21], including quality checks, and trimming of the raw reads by FastQC v0.11.9 and Trimmomatic v0.36. Contigs were assembled from the trimmed data using SPAdes [22], followed by the tool “Filter SPAdes repeats.” In silico identification of acquired antimicrobial resistance genes, virulence genes, plasmids, and clonal analysis were carried out using dedicated tools (MLST v.2.0, ResFinder v.3.2, Virulence Finder v.2021-03-01.1, Plasmid Finder v.2020.11.19, ABRICATE, SISTR) available at the Center for Genomic Epidemiology (http://www.genomicepidemiology.org/) and by the Basic Local Alignment Search Tool suite (https://blast.ncbi.nlm.nih.gov/Blast.cgi).

The freely available computational tool, PHASTER [23], was used to identify prophages and analyze the genomes in March 2023. Furthermore, a further analysis was performed on the putative plasmid contigs assembled using plasmid SPAdes v.3.15.0 [24] and annotated using the Rapid Annotations using Subsystems Technology (RAST) server [25].

In addition, raw reads were uploaded to EnteroBase (http://enterobase.warwick.ac.uk/species/index/senterica) under accession Barcode SAL_QB2921AA.

Phylogeny of *Salmonella* was examined by performing a search in Enterobase to identify all *Salmonella* isolates with the same sequence type (http://enterobase.warwick.ac.uk/species/senterica/search_strains) and GrapeTree was used to construct a rapid neighbor joining (RapidNJ) minimum spanning tree based on the core genome multi-locus sequence typing (cgMLST) V1 + hierarchical clustering (HierCC) V1 scheme in EnteroBase [26, 27].

### 2.4. Detection of Antibiotic Resistance Genes

Polymerase chain reaction (PCR) was used to screen for genes associated with resistance to beta-lactams (*bla*_TEM_, *bla*_SHV_, *bla*_CTX-M_, *bla*_CMY-1_, and *bla*_CMY-2_), carbapenems (*bla*_IMP_, *bla*_OXA-48_-like, *bla*_NDM_, and *bla*_KPC)_, colistin (*mcr*-1, *mcr*-2, *mcr*-3, *mcr*-4, and *mcr*-5), tetracyclines (*tet*A, *tet*B, *tet*C, *tet*L, *tet*M, and *tet*K), sulfonamides (*sul*1, *sul*2, and *sul*3), and aminoglycosides (*aac*C1, *aac*3, *aac*A4, *aph*A6, *arm*A, *rmt*B, *rmt*C, and *rmt*F) in Gram-negative isolates, with the exception of *Salmonella* isolate, as previously described [13]. The PCR protocols for Gram-positive isolates were conducted to detect genes associated with resistance to quinupristin/dalfopristin (*vg*A, *msr*C, *vat*D, *vgb*B, *vgb*A, *erm*B, and *vat*E), vancomycin (*van*A, *van*B, *van*C1, *van*C2, *van*D, *van*G, *van*M, and *van*N), linezolid (*cfr*, *cfr*B, *cfr*D, *optr*A, and *poxt*A), nitrofurantoin (*nfs*A, *nfs*B), tetracyclines (*tet*A, *tet*B, *tet*C, *tet*L, *tet*K, and *tet*M), macrolides (*erm*A, *erm*B, *erm*C, *erm*TR, and *mef*A/E), quinolones (*gyr*A), and beta-lactams (PBP1a, PBP2x, and PBP2b), as previously described [13].

## 3. Results

**Figure 2:**
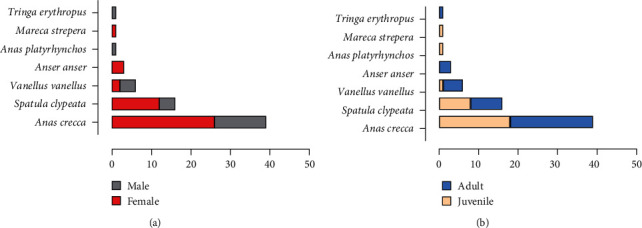
The number of sampled individuals according to sex (a) and age classes (b), as defined by trained ornithologists.

A total of 39 Eurasian teals (*Anas crecca*), 16 Northern shoveler (*Spatula clypeata*), six Northern lapwings (*Vanellus vanellus*), three graylag geese (*Anser anser*), one mallard (*Anas platyrhynchos*), one gadwall (*Mareca strepera*), and one spotted redshank (*Tringa erythropus*) were sampled. Of these, 65.7% (44/67) were identified as females and 34.3% (23/67) as males (Figure 2(a)). According to age classes as defined by trained ornithologists, 58% (39/67) of the birds were aged as adults, and 42% (28/67) were juveniles (first calendar year) (Figure 2(b)). All the samples used for this study resulted negative for AI.

**Figure 3:**
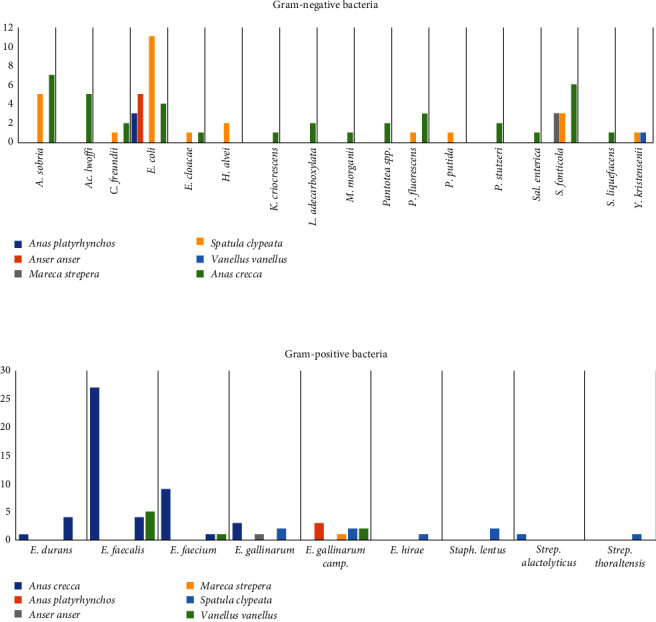
Gram-negative and Gram-positive bacteria isolated from wild aquatic birds sampled in Northern Italy.

Bacteriological investigations allowed to isolate 157 strains from 67 CS collected from the abovementioned population of interest.  Table 1 and Figure 3 summarize all the bacterial strains detected and the animal species involved.

**Figure 4:**
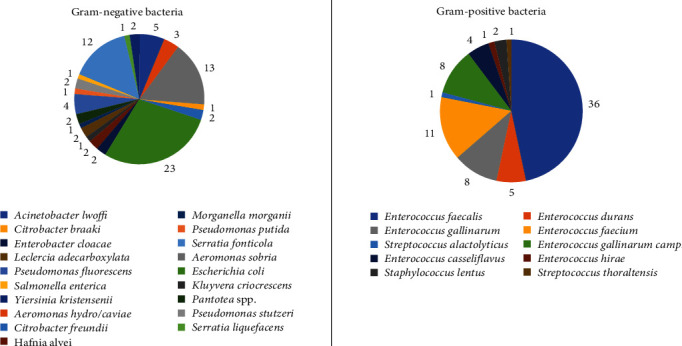
Distribution of bacterial species obtained from wild aquatic birds sampled in Northern Italy.

In detail, 80 isolates were Gram-negative, while 77 isolates were Gram-positive (Figure 4).

The most frequent isolates were *Enterococcus faecalis* (36/157; 22.9%) and *E. coli* (23/157; 14.6%). Overall, 77 isolates (77/157; 49%) showed phenotypic resistance to at least one antibiotic, and eight isolates (8/157; 5%) were classified as multi-drug resistant (MDR) bacteria showing resistance to at least three different classes of antibiotics. In addition, 12 Gram-negative (12/80; 15%) and seven (7/77; 9%) Gram-positive bacteria were found to be resistant to at least one critically important antibiotic. Among them, seven bacteria were MDR too. The details concerning the phenotypic and genotypic profiles are reported in Tables 2–4.

Whole genome sequencing has allowed to describe the serovar *Salmonella* Braenderup ST22 (Barcode: SAL_QB2921AA) with a single plasmid, which was identified as an IncI1. The plasmid sequence contained the mediator of hyperadherence YidE and resistance factors to copper, mercury, and zinc (CcmF, CcmH, CopD, CopC, CueO, and ZitB). In addition, ABRIcate identified the chromosomally located antibiotic resistance gene *aac*(6′)-Iaa, which is reported to confer resistance to amikacin and tobramycin [28].

Eleven prophages were detected by the PHASTER web server and two of these were classified as intact. No resistance or virulence genes were identified within the prophage's sequences.

**Figure 5:**
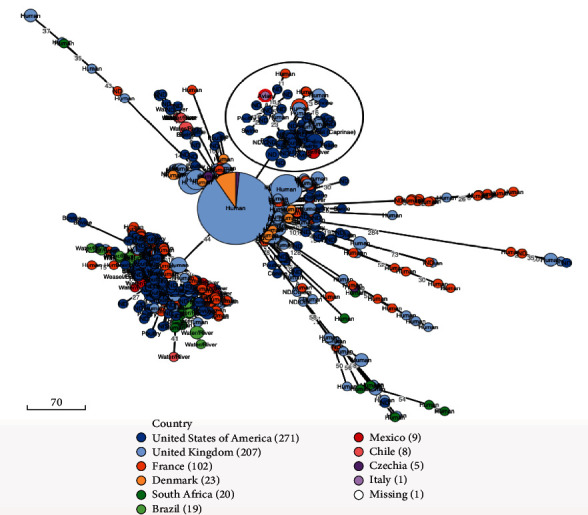
Phylogenetic tree showing the relatedness of *Salmonella enterica* serovar Braenderup isolate SAL_QB2921AA and *Salmonella* Braenderup ST22 isolates identified in Enterobase and collected during 2021 and 2022. GrapeTree was used to construct a rapid neighbor-joining (RapidNJ) minimum spanning tree based on the core genome multi-locus sequence typing (cgMLST) V1 + hierarchical clustering (HierCC) V1 scheme. The SAL_QB2921AA isolate is highlighted with a red circle with the cluster circled in black. The source type of each isolate is written on node and where is not available is indicated wit ND. Scale bar indicates the number of cgMLST allelic differences.

The phylogenetic relationship with all ST22 sourced in 2021 and 2022 showed SAL_QB2921AA belonged to large clade of isolates, of which the vast majority (351/666; 52.7%) were associated with human salmonellosis cases. Additionally, other isolates were identified from poultry (48/666; 7.2%), environment (plant, soil, or water) (46/666; 6.9%), livestock (36/666; 5.4%), food (8/666; 1.2%), wild animals (8/666; 1.2%), and companion animals (1/666;0.1%) (Figure 5). SAL_QB2921AA was placed in a cluster containing 78 isolates from the United States of America (USA), France, United Kingdom (UK), and Mexico. In detail, the isolates from France and UK were associated with human samples, while the isolates from Mexico were identified from environmental samples, and the isolates from USA were linked to livestock, poultry, wild animals, and other unspecified source types. The most closely related isolate (SAL_XC6709AA_AS) showed eight cgMLST allelic differences to SAL_QB2921AA, and it was detected from unspecified source type in North America during 2022.

## 4. Discussion

The results of our study provide additional information on antibiotic resistant bacteria isolated from wild aquatic birds in Italy. In detail, this report provides the first phenotypic and genotypic analysis combined to describe antibiotic resistance in Gram-positive and Gram-negative bacteria isolated from hunted wild aquatic birds, such as graylag goose (*A. anser*), gadwall (*M. strepera*), Northern shovelers (*S. clypeata*), and Northern lapwing (*V. vanellus*). Indeed, similar available studies are mainly focused on Eurasian teal (*A. crecca*), mallard (*A. platyrhynchos*), and other families of wild birds (Columbidae, Corvidae, Falconidae, Stringidae, and Laridae) [29–37]. Additionally, the investigations carried out in abovementioned studies are mostly focused on phenotypic or genotypic analysis of *E. coli* and/or *Enterococcus* spp.

*E. coli* isolates identified in this study involve waterfowl for which no data on resistant bacteria are present in the literature, namely gadwall (*S. clypeata*), Eurasian teal (*A. crecca*), and graylag goose (*A. anser*). Comparing the results hereby obtained for *E. coli* with those of other manuscripts focused on aquatic birds, the same phenotypic resistance to cefoxitin and ESBL was found in specimens recovered from a rehabilitation center in Northern Italy and from carcasses examined in Netherlands, while colistin-resistant isolates have previously been described only in strains from free-living animals in Poland and Spain [30, 34, 37, 38]. The resistance to piperacillin/tazobactam reported in this study has not been documented in other *E. coli* strains isolated from wild aquatic birds surveyed elsewhere. Information regarding piperacillin/tazobactam resistance in *E. coli* is of interest considering that this molecule is used for empirical treatments of various infections in human medicine and resistant strains have been increasing in recent years [39]. Regarding the resistance genes related to the phenotypic resistance described and identified in this study, the *bla*_TEM_ gene has been reported in studies involving aquatic bird species performed with culture-independent methods in the USA and Australia [40, 41], while *bla*_CMY-1_, *mcr*-3, and *mcr*-4 have been described for the first time in the category of animals under investigation. These aforementioned genes have previously been described in other species of domestic and wild animals, as well as in various water sources [42–48].

Regarding the Enterobacterales strains other than *E. coli* and the Pseudomonadales strains identified in the study, it was possible to describe profiles of colistin resistance for the first time in water bird species not including seagulls. The resistance to third-generation cephalosporins and carbapenems, documented in this study, has been described in wild bird species other than those studied, in animals sampled in a wildlife recovery center in Spain and in various studies investigating the role of gulls and white storks as reservoirs of antibiotic resistance [35, 38, 49]. Indeed, the related carbapenem resistance genes described in this study have also been previously documented in gulls in Spain [32].

Additionally, it was possible to describe resistance profiles not yet reported in aquatic birds. In a recent study conducted on animals of a wildlife recovery center in Southern Italy, resistance profiles of *Serratia* spp., *Citrobacter* spp., *Enterobacter* spp., and *Pseudomonas* spp. were described [50]. In our study, it was also possible to characterize other species such as *Aeromonas sobria*, *Aeromonas hydro/caviae*, *Leclercia adecarboxylata*, and *Acinetobacter lwoffii*. *Aeromonas* spp. is a bacterial species of interest given its emergence as a food-borne pathogen implicated in human gastroenteritis and extraintestinal diseases [51]. Furthermore, *Aeromonas* spp. has previously been discovered in food, animals, and birds, and it has been described in surface water in Italy [13, 51, 52]. Indeed, the aquatic environment is considered a potential vehicle for human infections with aeromonads [51].

*Leclercia adecarboxilata* has been also defined as an emerging human pathogen with the potential to cause severe infection in immunocompromised patients [53]. In this view, the resistance profile of this bacterium isolated in aquatic context appears to be relevant.

*A. lwoffii* was previously described in a variety of environments (i.e., animals, human skin and gut, and water sources) [54] and has been increasingly reported as a hospital pathogen responsible of septicemia, pneumonia, meningitis, urinary tract infections, skin and wound infections, and gastroenteritis [55, 56].

Regarding the results on Enterococci, the resistance to vancomycin, beta-lactams, and lincosamides reported in this study has been previously described in different wild birds from Australia, Sweden, Poland, Portugal, Spain, and Italy [35, 57–62]. On the contrary, for the first time, the linezolid resistance and the related *optr*A gene were described in isolates from hunted aquatic birds. The *optr*A gene was first identified in *E. faecalis* and *E. faecium* strains from humans and food-producing animals in China [63]. However, the *optr*A gene has also been detected in *E. gallinarum* and *E. hirae* of human origin in China and from pigs in Italy [63]. The dispersion of transferable oxazolidinone resistance genes among enterococci of different ecosystems poses a serious problem to human health [64].

On this topic, another transferable *poxt*A oxazolidinone resistance gene, previously described in methicillin resistant *Staphylococcus aureus* isolated in Italy [63], has been hereby identified in a phenotypically resistant *Staph. lentus*. This isolate was found to be MDR, as were other strains, including *Streptococcus thoraltensis* and *Streptococcus alactolyticus*.

The *Staphylococcus lentus* (part of the *Staphylococcus sciuri* group) is an uncommon and opportunistic pathogen, being associated with urinary tract infections in humans, mink (*Mustela vison*), and companion animals [65–67]. It has recently been described in a case of pyometra in wild European hedgehogs, and it has been reported in the feces of wild turkeys [68, 69].

*Streptococcus thoraltensis* and *S. alactolyticus* have previously been described in feces of rabbits and in humans with infections without any relevant antibiotic resistance profiles [70–72].

Another interesting finding is the presence of vancomycin resistance associated with *van*G and *van*M genes, providing new information on this occurrence in Northern Italy. Indeed, the van operons most investigated in previous studies are *van*A and *van*B [73].

In light of these findings, taking a more comprehensive view of intestinal bacterial species, including not only *E. coli*, *E. faecium*, and *E. faecalis*, could allow to obtain a broader understanding of resistance profiles in the aquatic niches. Indeed, aquatic systems have been defined as major transmission routes between wildlife and humans [74, 75]. Therefore, investigating resistance profiles in aquatic birds, including migratory birds, can provide information regarding environmental contamination from emerging and relevant resistant bacteria for public health, as shown by the data reported in this study.

In this way, it has been possible to identify resistance profiles related to critically important antibiotics for human medicine (linezolid, vancomycin, carbapenems, third-generation cephalosporins, and fluoroquinolones), which are considered the last resources for treating of multiresistant bacteria [17]. These types of resistance have been identified in MDR bacteria and in microorganisms that are included in the WHO's list of priority pathogens (carbapenem-resistant Enterobacterales and third-generation cephalosporin-resistant Enterobacterales), as similarly observed for seagulls [35].

The resistance results can be interpreted as arising from the ecological behavior of the species involved. Indeed, ducks filter water and sediments to trap plant and animal material. It is possible that ducks ingest large numbers of bacteria while dabbling, as previously suggested by other authors [40].

In this regard, wild birds have been defined as potential carriers not only of antibiotic-resistant bacteria but also of other relevant or zoonotic pathogens that can affect animals, humans, and the environment [76–78]. In many studies, they have been investigated for the presence of food-borne pathogens, such as *Salmonella spp*., in their intestines [31].

*Salmonella* spp. has previously been described in various species of free-living birds in Germany, Spain, and Poland, as well as in species housed in rehabilitation centers in Italy [29, 50, 79, 80]. The presence of *Salmonella* spp. has also been investigated in healthy game birds in UK [9], as well as in healthy migratory birds, including hunted waterfowl in Finland, Spain, Bulgaria, and Texas [31, 33, 81, 82]. The SAL_QB2921AA isolate identified in this study is the only *Salmonella* Braenderup ST22 isolate sourced from aquatic birds available in literature. A similar strain was recently investigated by European Food Safety Authority for a multicountry outbreak presumed to be linked to imported melons in Europe during 2021 [83]. However, the phylogenetic analysis of our isolate revealed a closer correlation with other isolates identified in USA during an outbreak involving 75 people, but with potential foods linked to illness still unknown (https://www.fda.gov/food/outbreaks-foodborne-illness/investigations-foodborne-illness-outbreaks). These results underscore the importance of providing useful genetic information that can allow to identify possible routes of pathogen spread in the environment [84]. In fact, migratory species have been considered relevant for their ability to cover long distances and link different countries and environments [5, 74]. As hypothesized for antibiotic-resistant bacteria, the ability of these animals to be carriers of food-borne pathogens is closely linked to their ecological behavior. Eurasian teal (*A. crecca*), the hunted species in which *Salmonella* ST22 was isolated, is a long-distance migratory dabbling duck that moves along the Black Sea-Mediterranean flyway for breeding in Siberia and Northern Europe and wintering in Western Europe, including Italy [85]. It usually inhabits low-anthropized wetlands, such as the ones of the study area, where it can encounter also pathogenic bacteria spread from inefficiently treated municipal sewage or agricultural practices [86]. This further highlights the potential health risks for hunters and consumers related to the consumption of wild game meat, as previously suggested by other authors [9, 33]. Good hygiene during game bird handling, storing the game bird meat frozen, and proper heat treatment before consuming remain important measures to reduce the risk of human exposure to food-borne diseases [33].

## 5. Conclusions

The migratory aquatic birds may serve as a relevant carrier of critically important antibiotic resistant bacteria and zoonotic food-borne pathogens which may have a potential impact on public health [33, 87]. The results of this study provide new data about the environmental contamination of antimicrobial resistance and food-borne pathogens useful for public health specialists.

## Figures and Tables

**Table 1:** Bacterial species isolated from wild aquatic birds sampled in Northern Italy.

Bacterial species	Total
*Acinetobacter lwoffi*	5
*Aeromonas hydrophila/caviae*	3
*Aeromonas sobria*	13
*Citrobacter braaki*	1
*Citrobacter freundii*	2
*Enterococcus casseliflavus*	4
*Enterococcus durans*	5
*Enterococcus gallinarum*	8
*Enterococcus gallinarum ^*∗*^*	7
*Escherichia coli*	23
*Enterococcus faecalis*	36
*Enterococcus faecium*	11
*Enterococcu shirae*	1
*Enterobacter cloacae*	2
*Hafnia alvei*	2
*Kluyvera criocrescens*	1
*Leclercia adecarboxylata*	2
*Morganella morganii*	1
*Pantotea* spp.	2
*Pseudomonas fluorescens*	4
*Pseudomonas putida*	1
*Pseudomonas stutzeri*	2
*Salmonella enterica*	1
*Serratia fonticola*	12
*Serratia liquefacens*	1
*Staphylococcus lentus*	2
*Streptococcus alactolyticus*	1
*Streptococcus thoraltensis*	1
*Yiesinia kristensenii*	2
Total	157

^*∗*^CAMP test positive isolates by Vitek 2 system (Biomerieux, France).

**Table 2:** Multi-drug resistant (MDR) bacteria isolated from wild aquatic birds sampled in Northern Italy.

Bacterium	Animal	Multidrug resistant phenotype	Number of classes of antibiotics	Number of isolates
*S. fonticola*	*Anas crecca*	AK CAZ CN ETP FOX	3	1
*E. hirae*	*Spatula clypeata*	AMP LNZ QD TEIC VAN	4	1
*E.casseliflavus*	*Spatula clypeata*	LNZ QD TEIC	3	1
*E.gallinarum camp*.	*Spatula clypeata*	AMP LNZ QD TEIC	4	1
*Staph. lentus*	*Spatula clypeata*	CLIN DAP LNZ OX RD SXT TEIC TET VAN	8	1
*Staph. lentus*	*Spatula clypeata*	CLIN OX STX	3	1
*Strep. alactolyticus*	*Anas crecca*	BEN CLIN CTX CRO ERY LEV MXF VAN	5	1
*Strep. thoralensis*	*Spatula clypeata*	BEN CLIN CTX ERY LEV TET	5	1

AMP, ampicillin; AK, amikacin; BEN, benzypenicillin; CAZ, ceftazidime; CLIN, clindamycin; CN, gentamycin; CTX, cefotaxime; CRO, ceftriaxone; DAP, daptomycin; ERY, erythromycin; ETP, ertapenem; FOX, cefoxitin; LEV, levofloxacin; LNZ, linezolid; MXF, moxifloxacine; OX, oxacillin; QD, quinupristin/dalfopristin; RD, rifampicin; SXT, trimethoprim/sulfamethoxazole; TEIC, teicoplanin; TET, tetracycline; and VAN, vancomycin.

**Table 3:** Phenotypic and genotypic resistance profiles of Gram-negative bacteria isolated from wild aquatic birds sampled in Northern Italy.

Resistant isolates	Animal	Antibiotics	Resistance genes
*A. sobria*	*Anas crecca*	CS	*mcr*-3, *mcr*-4
	*Anas crecca*	TZP MRP	—
	*Anas crecca*	MRP	—
	*Spatula clypeata*	TZP CS	—
	*Anas crecca*	ESBL	—
	*Spatula clypeata*	ESBL	*bla*_TEM_

*A. hydrophila/caviae*	*Anas crecca*	CS	*mcr*-4
	*Spatula clypeata*	ESBL	*bla*_TEM_
	*Spatula clypeata*	CS	*mcr*-2, *mcr*-3, *mcr*-4
	*Anser anser*	CS	*mcr*-2, *mcr*-4

*Ac. lwoffi*	*Anas crecca*	CS ESBL	*mcr*-4, _*bla*CMY-2_
	*Anas crecca*	ESBL	—

*C. braaki*	*Spatula clypeata*	FOX ^*∗*^ CS	ESBL, *mcr*-4

*Ent. cloacae*	*Spatula clypeata*	FOX ^*∗*^ CAZ ^*∗*^	—
	*Anas crecca*	FOX ^*∗*^ CAZ ^*∗*^ ESBL ETP	*bla*_NDM_, *bla*_KPC_

*E. coli*	*Spatula clypeata*	TZP	—
	*Spatula clypeata*	ESBL	*bla*_CMY-1_, *bla*_TEM_
	*Spatula clypeata*	FOX	—
	*Spatula clypeata*	ESBL	*bla*_CMY-1_, *bla*_TEM_
	*Anas crecca*	ESBL	—
	*Spatula clypeata*	ESBL	—
	*Spatula clypeata*	ESBL	—
	*Spatula clypeata*	ESBL	—
	*Anas crecca*	ESBL	*bla*_TEM_, *bla*_CMY-1_
	*Anser anser*	ESBL CS	*bla*_TEM_, *bla*_CMY-1_, *mcr*-3, *mcr*-4
	*Anser anser*	ESBL	*bla*_TEM_, *bla*_CMY-1_
	*Anser anser*	ESBL	*bla*_TEM_, *bla*_CMY-1_
	*Anser anser*	ESBL	*bla*_CMY-1_
	*Anser anser*	ESBL	*bla*_CMY-1_

*H. alvei*	*Spatula clypeata*	ESBL	—

*K. criocrescens*	*Spatula clypeata*	ESBL	*bla*_TEM_

*L. adecarboxylata*	*Anas crecca*	FOX ESBL	*bla*_SHV_, *bla*_TEM_

*Morg. morganii*	*Anas crecca*	ETP	—

*Pantotea* spp.	*Anas crecca*	FOX CAZ ETP ESBL	*bla*_CMY-1_, *bla*_CMY-2_, *bla*_NDM_, *bla*_KPC_

*P. fluorescens*	*Spatula clypeata*	ESBL	—
	*Anas crecca*	CS ESBL	*mcr*-2, *mcr*-4
	*Anas crecca*	ESBL	—
	*Anas crecca*	ESBL	—

*P. putida*	*Spatula clypeata*	ESBL	—

*P. stutzeri*	*Anas crecca*	CS	*mcr*-4
	*Anas crecca*	ESBL	—

*S. fonticola*	*Anas crecca*	FOX	—
	*Anas crecca*	ESBL	—
	*Spatula clypeata*	FOX	—
	*Anas crecca*	AK FOX CAZ CN ETP	*bla*_NDM_, *bla*_KPC_
	*Mareca strepera*	ESBL	*bla*_CTX-M_
	*Mareca strepera*	ESBL	*bla*_CTX-M_

*Salmonella enterica*	*Anas crecca*	—	*aac*(6′)-Iaa

*S. liquefacens*	*Anas crecca*	FOX	—

AK, amikacin; CAZ, ceftazidime; CN, gentamycin; CS, colistin; ESBL, extended-spectrum beta-lactamase; ETP, ertapenem; FOX, cefoxitin; MRP, meropenem; TZP, piperacillin/tazobactam; and  ^*∗*^, intrinsic resistance.

**Table 4:** Phenotypic and genotypic resistance profiles of Gram-positive bacteria isolated from wild aquatic birds sampled in Northern Italy.

Resistant isolates	Animal	Antibiotics	Resistance genes
*E. casseliflavus*	*Anas crecca*	QD	*vat*D, *vat*E, *vg*A
	*Spatula clypeata*	QD	—
	*Spatula clypeata*	QD LNZ TEIC	*vat*D, *vg*A, *vgb*B
	*Vanellus vanellus*	QD	*vat*D, *msr*C
	*Vanellus vanellus*	QD	*vg*A, *msr*C

*E. durans*	*Anas crecca*	QD	*vat*E, *msr*C

*E. faecalis*	*Anas crecca*	DAP	—

*E. faecium*	*Anas crecca*	QD	—
	*Anas crecca*	QD	*vat*E, *msr*C
	*Anas crecca*	QD	*vat*D, *msr*C
	*Vanellus vanellus*	QD	*vat*E, *vg*A, *msr*C

*E. gallinarum*	*Anas crecca*	QD	—
	*Anas crecca*	QD	*vat*D, *vg*A
	*Spatula clypeata*	QD	—
	*Spatula clypeata*	QD	*erm*B, *msr*C, *vat*E, *vgb*A
	*Anser anser*	QD	*vgb*B
	*Anas crecca*	QD	*msr*C, *vat*D, *vg*A
	*Vanellus vanellus*	QD	*msr*C, *vat*D, *vg*A
	*Vanellus vanellus*	QD	*vat*D, *msr*C

*E. gallinarum ^*∗*^*	*Mareca strepera*	QD	*erm*B, *msr*C, *vga*B
	*Mareca strepera*	QD	*erm*B, *vga*B
	*Anas platyrhynchos*	QD	*erm*B, *vat*E, *vg*A
	*Anas platyrhynchos*	QD	*erm*B, *vat*E, *vg*A, *vga*B
	*Anas platyrhynchos*	AMP LNZ QD TEIC	*msr*C, *optr*A, *van*C1/C2, *van*M, *van*G, *vga*B

*E. hirae*	*Spatula clypeata*	AMP LNZ QD TEIC VAN	*optr*A, *van*M, *vgb*B

*Staph. lentus*	*Spatula clypeata*	CLIN DAP LNZ OX RD STX TEIC TET VAN	*Inu*A, *poxt*A, *tet*K, *van*D
	*Spatula clypeata*	CLIN OX STX	*sul*2

*Strep. thoraltensis*	*Spatula clypeata*	BEN CLIN CTX ERY LEV TET	*gyr*A, *Inu*A, *mef*A/E

*Strep. alactolyticus*	*Anas crecca*	BEN CTX CRO CLIN ERY LEV MXF VAN	*erm*B, *gyr*A, *Inu*A, *mef*A/E

AMP, ampicillin; BEN, benzypenicillin; CLIN, clindamycin; CTX, cefotaxime; CRO, ceftriaxone; DAP, daptomycin; ERY, erythromycin; LEV, levofloxacin; LNZ, linezolid; MXF, moxifloxacine; OX, oxacillin; QD, quinupristin/dalfopristin; RD, rifampicin; SXT, trimethoprim/sulfamethoxazole; TEIC, teicoplanin; TET, tetracycline; VAN, vancomycin; and  ^*∗*^CAMP test positive isolates by Vitek 2 system (Biomerieux, France).

## Data Availability

The data used to support the findings of this study are included within the article.
